# Lone Pair…π Contacts and Structure Signatures of r(UNCG) Tetraloops, Z-Turns, and Z-Steps: A WebFR3D Survey

**DOI:** 10.3390/molecules27144365

**Published:** 2022-07-07

**Authors:** Craig L. Zirbel, Pascal Auffinger

**Affiliations:** 1 Department of Mathematics and Statistics, Bowling Green State University, Bowling Green, OH 43403, USA; zirbel@bgsu.edu; 2 Architecture et Réactivité de l’ARN, UPR 9002, Institut de Biologie Moléculaire et Cellulaire du CNRS, Université de Strasbourg, 67084 Strasbourg, France

**Keywords:** nucleic acid, X-ray, cryo-EM, NMR, molecular dynamics simulations, structure mining, isosteric, isostructural

## Abstract

Z-DNA and Z-RNA have long appeared as oddities to nucleic acid scientists. However, their Z-step constituents are recurrently observed in all types of nucleic acid systems including ribosomes. Z-steps are NpN steps that are isostructural to Z-DNA CpG steps. Among their structural features, Z-steps are characterized by the presence of a lone pair…π contact that involves the stacking of the ribose O4′ atom of the first nucleotide with the 3′-face of the second nucleotide. Recently, it has been documented that the CpG step of the ubiquitous r(UNCG) tetraloops is a Z-step. Accordingly, such r(UNCG) conformations were called Z-turns. It has also been recognized that an r(GAAA) tetraloop in appropriate conditions can shapeshift to an unusual Z-turn conformation embedding an ApA Z-step. In this report, we explore the multiplicity of RNA motifs based on Z-steps by using the WebFR3D tool to which we added functionalities to be able to retrieve motifs containing lone pair…π contacts. Many examples that underscore the diversity and universality of these motifs are provided as well as tutorial guidance on using WebFR3D. In addition, this study provides an extensive survey of crystallographic, cryo-EM, NMR, and molecular dynamics studies on r(UNCG) tetraloops with a critical view on how to conduct database searches and exploit their results.

## 1. Introduction

Defining and naming structural motifs is key to better comprehending and manipulating nucleic acid systems and is a root of structural ontology [1,2]. A significant part of nucleic acid motifs has already been categorized with various levels of precision. Efficient tools to search these motifs in PDB structures are currently accessible [3,4,5,6,7,8,9,10]. However, some motifs have not yet been defined with the finest possible levels of detail. Henceforth, the structural features of a few of the best-known and most essential of these motifs can still surprise us. We illustrate this by focusing on r(UNCG) tetraloops that are ubiquitous in RNA systems [11].

A Background section summarizes the main characteristics of these tetraloops and how their structural signature evolved and was recently augmented by the discovery of an embedded Z-step that is isostructural to CpG steps in Z-DNA [12,13]. In the same section, we discuss the importance of lone pair…π or lp…π contacts in the context of Z-steps and r(UNCG) tetraloops and address their structural and energetic features. In nucleic acids, lp…π contacts are defined as involving the stacking of an oxygen atom with a nucleobase [12,14,15,16,17]. To conclude the Background section, we survey molecular dynamics (MD) simulations related to r(UNCG) and Z-DNA systems that contain Z-steps and discuss current challenges that are related to obtaining stable dynamical models.

In the Results section, we describe a novel WebFR3D [5] implementation of symbolic constraints allowing one to search lp…π contacts in RNA. To the best of our knowledge, WebFR3D is currently the only publicly available structural search tool that embeds such functionality. We illustrate how these new WebFR3D constraints can be used to search PDB structures for Z-steps, for exploring Z-turns, and for defining effective structural signatures for r(UNCG) tetraloops and their variants. Obtaining such signatures is of great interest for providing sound structural references that can be used to better tune and validate MD simulations. We conclude by discussing future evolutions of the service that will incorporate the ability to search for Z-steps in DNA and to search for motifs that include modified nucleotides.

## 2. Background: r(UNCG) Tetraloops and Lone Pair…π Contacts

### 2.1. The Structural Signature of r(UNCG) Tetraloops Has Evolved

**Figure 1:**
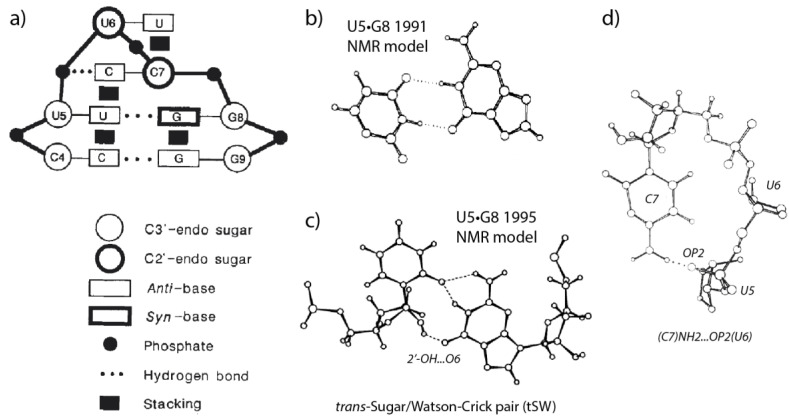
**Early and revised r(U_5_UCG_8_) NMR model structures.** (**a**) First NMR structural signature (adapted with permission from [18]). (**b**) First model of the U•G pair in an r(UUCG) loop (adapted with permission from [19]). (**c**) The preceding model was revised in 1995 to lead to the correct trans-Sugar/Watson-Crick (tSW) base pair arrangement (adapted with permission from [22]). Note that a C•G tSW pair would be perfectly isosteric to the U•G tSW pair. (**d**) A C7…U5 base…phosphate interaction, annotated as 8BPh, is part of the r(UUCG) tetraloop signature (adapted with permission from [18]).

The first r(UUCG) tetraloop NMR structure was characterized some 30 years ago [18,19]. This 1990 structure established that the r(U_5_UCG_8_) loop embeds a reverse-wobble U_5_•G_8_ pair with the guanine adopting a *syn* conformation, with a cytosine…phosphate contact and with extensive base stacking (Figure 1). The structural model also involved a *C2′-endo* conformation for U6 and C7 associated with a specific backbone dihedral angle sequence [18,19,20]. As a further r(UNCG) characteristic, the Watson-Crick edge of the U_6_ nucleobase is usually fully exposed to the solvent.

The r(UUU_7_G) NMR structure looks like that of the parent r(UUC_7_G) tetraloop. Along with r(GNRA) tetraloops, r(UNCG) loops are among the most stable RNA tetraloops while r(UUU_7_G) loops were found to be less stable [18,21]. The C7/U7 replacement, which leads to an apparent loss of the cytosine…phosphate contact (Figure 1d), was considered to be at the origin of the lower thermodynamic stability of the r(UUU_7_G) loop.

Some parts of the original r(UUCG) structure were revised in 1995 through improved NMR protocols [22]. The new model mainly differs in the hydrogen bonding pattern of the U_5_•G_8_ pair that was found to involve a 2′-OH group (Figure 1). The authors noted that the resonance rate of exchange of the 2′-OH hydrogen is like that of NH groups involved in Watson-Crick pairs. Thus, this 2′-OH forms a stable hydrogen bond. This contrasts with the fact that a majority of the 2′-OH resonances are unobservable given that the hydroxyl proton is usually in fast exchange with those of the solvent.

In a subsequent 2010 “high-resolution” NMR structure (PDBid: 2KOC), the tetraloop U•G pair was annotated as *trans*-wobble [23]. Additionally, based on NOESY water-RNA cross peaks, it was suggested that a water molecule was firmly associated with this base pair. Later, this base pair was annotated as *trans*-Sugar/Watson-Crick (tSW) based on the Leontis and Westhof base pair nomenclature [5,24,25].

The first X-ray structure of a r(UUCG) tetraloop was obtained in 2000 (PDBid: 1F7Y; resolution: 2.8 Å; [24]). The authors described failures related to early attempts to crystallize standalone r(UUCG) hairpins and identified a successful strategy that consisted in embedding the tetraloop in a larger RNA system, namely a 16S rRNA fragment complexed with a *Thermus thermophilus* S15 r-protein. The two r(UUCG) tetraloops embedded in the system were similar to those of the 1995 revised NMR structure [22,24].

**Figure 2:**
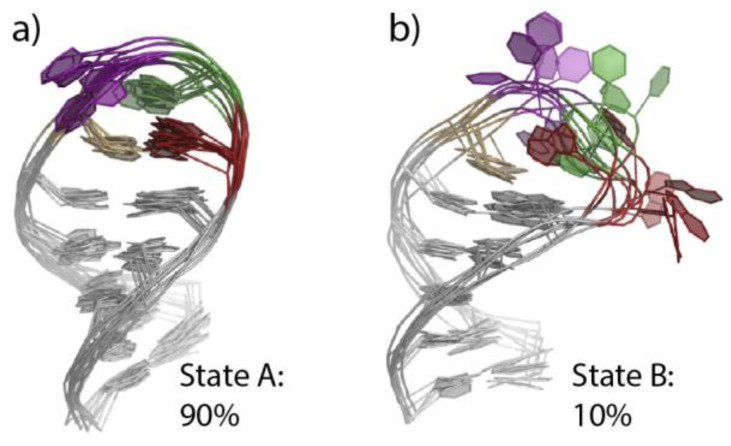
**Two NMR ensembles of an r(UUCG) tetraloop derived from eNOE experiments.** (**a**) Representative (random) conformations sampled from state A corresponding to a canonical fold. (**b**) Representative (random) conformations sampled from state B corresponding to less structured conformations. Note the fraying out of the guanine of the U•G pair. The PDBids of these NMR ensembles are 6BY4 and 6BY5. Figure adapted from [27].

More recently, NMR studies reported r(UNCG) conformational heterogeneity [25,26]. A study using exact nuclear Overhauser enhancement data (eNOEs) led to the characterization of a two-state r(UUCG) structural ensemble [26]. This two-state dynamic was explored through the integration of molecular dynamics (MD) simulations with eNOEs [27]. The dominant state (state A; 90%) corresponds to the consensus r(U_6_UCG_9_) structure, while the low-populated state B (~10%) is characterized by the absence of the U_6_•G_9_ tSW pair with C_8_ and G_9_ partially exposed to the solvent. However, the possibility that conformations differing from states A and B can be present was suggested (Figure 2). The advantage of combining NMR data with structure prediction and MD simulations is to be able to circumvent current MD simulation shortcomings and imprecisions to construct more reliable dynamic conformational ensembles [28,29]. MD simulation shortcomings are addressed in Section 2.3.

### 2.2. r(UNCG) Tetraloops, Although Called Z-Turns, Comprise a CpG Z-Step with a Lone Pair…π Contact

Up to recently, it has escaped the attention of most structural biologists except Richardson et al. [20], that the CpG step of r(UNCG) tetraloops adopt a conformation that is also found in CpG containing double-helical Z-DNA structures. Therefore, it is important to describe some of the main structural characteristics of these CpG steps.

Because they are characteristic of the Z-DNA zig-zag left-handed structure, Z-DNA CpG steps were named Z-steps [12,13]. By extension, any DNA or RNA NpN dinucleotide that adopts a conformation close to that of the Z-DNA CpG step is called a Z-step. The fact that almost all dinucleotide NpN sequences, when placed in the proper environment, can form Z-steps stresses the structural importance of this recurring nucleic acid motif [12,30]. Further, UNCG-like tetraloops and larger loops containing Z-steps are called Z-turns [13,31]. Rare examples of Z-turns with an r(GAAA) sequence featuring an ApA Z-step were identified in the core of ribosomes and in a few other RNAs [13,31]. They are discussed in Section 3.5.

**Figure 3:**
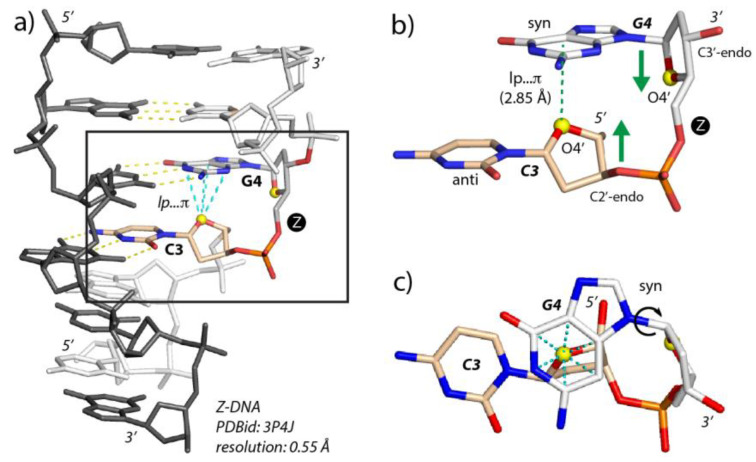
**A representative Z-DNA CpG step and associated lp…π contact.** (**a**) View of a 0.55 Å X-ray structure of a *d*(CpGpCpGpCpG)_2_ duplex [32]. A representative CpG segment is boxed and colored as in (**b**); the remaining nucleotides of this strand are in white. The second strand is in dark grey. The lp…π contacts and the base pair hydrogen bonds associated with the CpG segment are marked by cyan and yellow dashed lines, respectively. This structure contains six lp…π contacts, one for each CpG step. (**b**) Close-up of the CpG motif boxed in (**a**). The O4′ atoms are yellow; the cytosine (C) and guanine (G) carbons are wheat and white, respectively. The O4′ atom of C3 stacks with the 3′ face of G4. Note the characteristic Z-DNA alternation of *anti* and *syn* conformations [12]. The head-to-head orientation of the two sugar rings, common for Z-steps, is marked by green arrows. (**c**) Orthogonal view of b). In (**a**,**b**), the “Z” letter (circled with a black background) marks the occurrence of a Z-step.

The Z-DNA CpG Z-step conformation (Figure 3) involves a 3′-nucleotide in a *syn* conformation, a 5′-nucleotide (deoxy)ribose with a C2’-*endo* pucker, and an lp…π contact with an oxygen (O4′) to nucleobase (G) contact distance below 3.5 Å and sometimes close to 2.8 Å. Z-steps also embed a characteristic ribose head-to-head (*The head-to-tail versus head-to-head terms sometimes lead to confusion. Here we chose to name head-to-tail the ribose orientation as it occurs in a regular helical structure by analogy with elephants walking in a line and holding the tail ahead with their trunk; we named head-to-head the ribose orientation shown in Figure 3b that occurs in Z DNA and r(UNCG) CpG steps. Note that we wrongly used “head-to-tail” in [13]*) orientation. The 3.5 Å boundary was set by examining the oxygen to nucleobase plane distance histograms derived from various NpG steps in nucleic acids [12,13,17] although other authors preferred 4.0 Å boundaries [15].

The *syn* conformation of the CpG Z-step guanine implies a χ torsion angle value around the glycosidic bond of about 60° instead of 120° for the usual *anti* conformation [33,34]. One of the rarely noted peculiarities of a Z-step is that the Watson-Crick sites of these base pairs are aligned, resulting in a modest average 2° rotation for a CpG step in Z-DNA compared to a 60° rotation for a Z-DNA GpC step and a ≈ 35° rotation for any step in B-DNA.

The energetic contribution of the lp…π contact is not precisely known, although it is appreciated that the interaction is of a weak non-covalent type [14,17]. We established earlier that the origins of the short ≈2.8 Å contacts observed in X-ray structures cannot be explained by orbital effects as implied by the “lp…π interaction” terminology. Thus, we proposed that these “lp…π interactions” could be named “oxygen…π contacts” to avoid interpretation issues [35,36,37]. Given the weak non-covalent character of these interactions, it has been hypothesized that short lp…π contacts occur primarily in structurally strained motifs such as those found in Z-DNA and r(UNCG) tetraloops [17,37].

**Figure 4:**
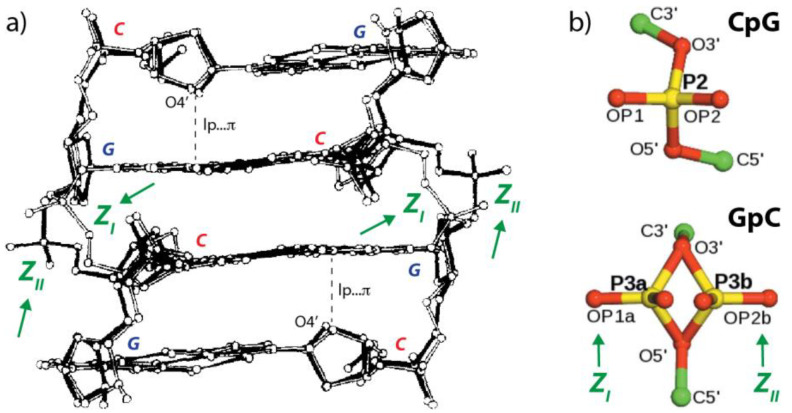
**Z_I_ and Z_II_ conformations.** (**a**) Superimposition of two Z-DNA *d*(CGCG)_2_ structures (PDBid: 1ZNA; resolution 1.5 Å) showing Z_I_ (light bonds) and Z_II_ (dark bonds) conformations (adapted with permission from [38]). Short lp…π contacts are marked by a dotted line. (**b**) Two characteristic conformations of a CpG (top) and a GpC (bottom) step extracted from a d(CGCGCGCGCGCG)2 dodecamer (PDBid: 4OCB; resolution: 0.75 Å). The CpG step displays mostly a single conformation while four of the five GpC steps of the dodecamer were modeled as alternate Z_I_ and Z_II_ conformations thanks to the use of phosphorous anomalous signals (adapted from [34]).

The dihedral angle variations of a CpG Z-step seem relatively limited when considering Z-DNA structures. The left-handed double-helical Z-DNA is constructed from alternating pyrimidine–purine (YpR) and purine–pyrimidine (RpY) steps. The YpR steps are characterized by a single backbone conformation whereas the RpY steps may adopt two distinct conformations known as Z_I_ and Z_II_ [38,39,40,41]. In the Z_I_ form the phosphate groups are shifted deeper inside the helix towards the groove and in the Z_II_ form the phosphate groups are rotated away from the groove (Figure 4). Sometimes, alternate conformations of phosphate groups are observed in high-resolution X-ray structures [34]. The consensus r(UNCG) dihedral backbone sequence is N**1a**U**1z**N**2**[C**6n**G**1a**N following the nomenclature established by Richardson et al. [20].

Two other local Z-DNA conformations were reported. These are the Z and Z’ forms that adopt a C3′-*endo* and an infrequent C4′-*exo* guanine sugar pucker, respectively [38,42]. Overall, the base pair orientation and stacking configuration seem not affected by changes in the guanine sugar pucker (Z, Z’) or the GpC backbone conformations (Z_I_, Z_II_).

### 2.3. r(UNCG) and Z-DNA Molecular Dynamics Simulation Challenges

Despite the apparent simplicity of these motifs, MD simulations of r(UNCG) tetraloops and Z-DNA fragments present many unresolved challenges that are related to an incomplete understanding of the forces at play in these systems [43,44,45,46,47,48,49,50,51,52,53]. Therefore, we provide next a survey of current MD simulation issues.

A recent report summarized some of these challenges for RNA tetraloops that could not all be resolved by recent parameterization efforts [54,55,56,57,58,59]. The characteristic r(UUCG) native structure is lost in ns to μs standard MD simulations or does not fold correctly because of at least two different effects. The first of those is excessive stabilization of unfolded single-stranded RNA structures by intramolecular base…phosphate and sugar…phosphate interactions. The second relates to the destabilization of the native folded state by underestimation of the native hydrogen bonds including the stem base pairing. The drift from the native structure was described as a progressive and undesired loss of key signature interactions.

Simulations using the newly developed DESRES force field documented several r(UUCG) unfolding-refolding events on a 20 μs time scale [55] but these were found to be sensitive to tiny changes in parameterization. For instance, changing the monovalent ion model suffices to completely lose the tetraloop native structure [54,60]. Other issues related to the use of the DESRES force field are described in references [54,55,57,60]. The authors of these studies conclude that they are not currently aware of any existing RNA force field that would accurately represent these tetraloop systems on μs time scales.

Thus, if the NMR data suggest the existence of a dominant and several minor states for the tetraloop, MD simulations must be able to sample these various populations and their variations related to changes in temperature and environmental conditions [61]. These issues will certainly push MD force fields and simulation protocols into further challenges and necessitate a finer understanding of the physico-chemistry underlying these phenomena.

Some attention was recently drawn to the parameterization of the lp…π contact constitutive of Z-steps, an issue that has never been fully addressed [37]. To this point, it is unknown to what extent a potentially imbalanced description of lp…π contacts causes MD simulations of Z-step-containing systems to ill-behave. However, two factors are worthy of consideration. The first is related to a misrepresentation of the Lennard-Jones or vdW parameters of the nucleobase atoms. It has been established that the vdW parameters of *sp^2^* carbon atoms attached to electron-withdrawing groups are largely overestimated by the Bondi tabulation established in 1964, a tabulation still in use [17,37]. A smaller effective *sp^2^* carbon vdW radius could explain the short lp…π contacts observed in Z-DNA and r(UNCG) loops. It can be noted that the current AMBER force-field versions use Lenard-Jones parameters for the nucleobase carbon atoms that are identical to those of phenyl ring carbons as noted in [37]. The second effect of importance for MD simulations is an overestimation of the repulsive part of the Lennard-Jones potential of current force fields when compared to potentials derived from high-level quantum-mechanics calculations. Although adapting the Lennard-Jones parameters is a difficult task needing significant reparameterization efforts, progress in this direction would certainly help to improve the quality of MD simulations of systems containing lp…π contacts and of biopolymers in general [37].

Besides r(UNCG) tetraloops, Z-DNA structures also represent a significant challenge for MD simulations [53,62,63,64,65]. This can be linked to an inappropriate representation of Z_I_/Z_II_ equilibrium states and has been recently addressed through backbone dihedral angle reparameterizations. Interestingly, these reports also mention a sensitivity of the explored substates to the monovalent ion parameters and water models suggesting that the entire molecular ecosystem must be modeled with the greatest possible accuracy to achieve a precise balance of interatomic forces. Given the presence of Z-steps in both Z-DNA and r(UNCG) tetraloops, progress in the simulation of these systems is definitely linked.

## 3. Results

### 3.1. Defining lp…π Contacts

As mentioned in [51], the native state definition of a structural model can have a dramatic impact on reported populations of folded states derived from MD simulations, NMR studies, or X-ray surveys. Therefore, the following aims at defining an operational structural signature for characterizing r(UNCG) tetraloops, associated Z-steps, and more generally Z-turns. For that, we start this section by defining lp…π contacts that are common to all these motifs.

Since a significant portion of dinucleotide fragments involving lp…π contacts are not of the Z-step type, it is important to first define an lp…π contact signature. The characteristic of these contacts is that the involved oxygen atom stacks on a nucleobase face with a contact distance to the nucleobase plane ≤ 3.5 Å [12,13,17]. We refer to the two faces as the 3′ and the 5′-face; for a definition of 3′-and 5′-nucleobase faces, see the Method Section.

The lp…π distance has also to be >2.0 Å to exclude rare “in-plane” contacts. Issues related to alternate conformations in database searches are described in [66]. Such conformations (with occupancies < 1.0) will be ignored in the following. Note that for generic lp…π contacts, the two nucleotides do not need to be consecutive or to belong to the same strand and the nucleobases do not need to be stacked.

### 3.2. Finding lp…π Contacts with WebFR3D

The WebFR3D server can search for lp…π interactions using text strings such as s3O4′ to indicate that the 3′-face of one nucleotide stacks with the O4′ atom of a second nucleotide. The first two characters, s3 or s5, mark a stacking with the nucleobase 3′ or 5′-face. The second part of the text string marks which backbone oxygen atom is involved in the stacking, for instance: OP1, OP2, O2′, O3′, O4′, or O5′ (nucleobase O2, O4, O6, and non-hydrogenated nitrogens are not considered). Note that the search string is directional. One may write that the 3′-face of nucleotide 1 (nt1) forms an s3O4′ interaction with the O4′ atom of nt2, or that nt2 forms a sO4′3 interaction with the 3′-face of nt1.

When searching for nucleotides forming lp…π contacts, various abbreviations can be used. For instance, “O” is generic for OP1, OP2, O2′, O3′, O4′, and O5′ backbone oxygen atoms. Thus, sO3 indicates stacking involving a backbone oxygen of nt1 and the 3′-face of nt2 while sO indicates stacking of a backbone oxygen atom on either the 3′ or 5′-face of nt2. Figure S1 (Supplementary Materials) shows the results of an sO search that identifies 246 lp…π contacts in the 7K00 ribosomal structure, 143 of which involve an O4′ atom (sO4′) and 62 involve an OP atom (sOP or sOP1/sOP2).

### 3.3. Z-Steps and Z_anti_-Steps Identified by WebFR3D

An ideal Z-step signature, as observed in Z-DNA and r(UNCG) tetraloops [12], involves two consecutive nucleotides where the 5′-nucleobase is in *anti*; the 5′-sugar is in *C2′-endo*; the 3′-nt is in *syn*, and the O4′ oxygen of the 5′-ribose stacks with the 3′-face of the 3′-nt to form an lp…π contact. Finally, in Z-DNA and r(UNCG) tetraloops, a ribose head-to-head orientation occurs (Figure 3b). As noted above, the consensus r(UNCG) dihedral backbone sequence for the CpG step is N**1a**U**1z**N**2**[C**6n**G**1a**N following the nomenclature established by Richardson et al. [20]. We note also that the head-to-head orientation of the two ribose is a consequence of the formation of an lp…π contact.

In the 7K00 ribosome structure at 1.98 Å resolution, eleven instances of Z-step motifs are identified that comprise six different sequences (Figure S2). In a larger search, most of the 16 possible r(NpN) sequences can be identified by WebFR3D when searching current representative sets of structures at different resolution thresholds. For instance: ApA, ApG, GpA, GpG, CpA, CpG, UpA, and UpG Z-steps are identified in structures with resolution ≤ 2.0 Å; ApC, ApU, CpU, UpC, and UpU Z-steps are identified in structures with resolutions between 2.0 Å and 2.5 Å, and GpC, GpU Z-steps are identified in structures with resolutions between 2.5 Å and 3.0 Å. This confirms earlier conjectures stating that Z-steps can involve any of the 16 NpN sequences except for CpC Z-steps in RNA structures [12,13]. Overall, we found that NpR are more frequent than NpY steps and without surprise that CpG is the most frequent Z-step in RNA as it is in DNA.

Z-steps with a 3′-nucleobase in *anti* that we called Z_anti_-steps [13]. They correspond to a Z-step subcategory that, along with many other variants, is not discussed here. All these variants can be identified by WebFR3D. Note that the *syn*/*anti* constraint is sometimes not very effective for the 3′-nt in a Z-step since the *χ* angles are in borderline regions. Therefore, it is suggested to use as an alternative an nt1…nt2 s53 stacking constraint when the *syn* constraint leads to questionable results. More precisely, s53 implies that the second base is turned and adopts a *syn* conformation. For searching Z_anti_-steps, where the second base is in *anti*, an s55 constraint can be used.

### 3.4. r(UNNG) Z-Turn Signatures Derived from X-ray and Cryo-EM Structures

**Figure 5:**
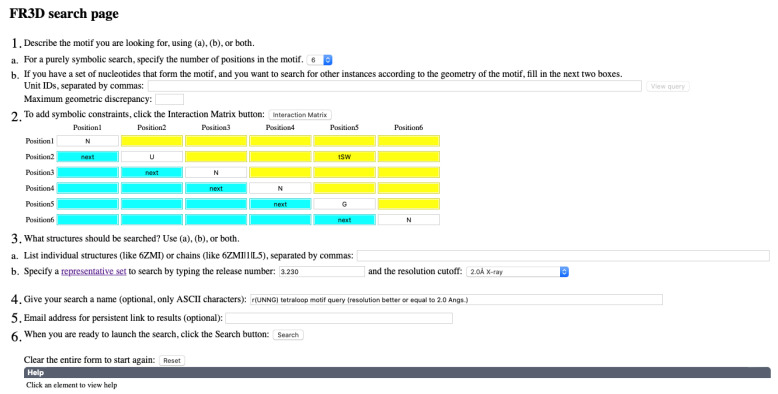

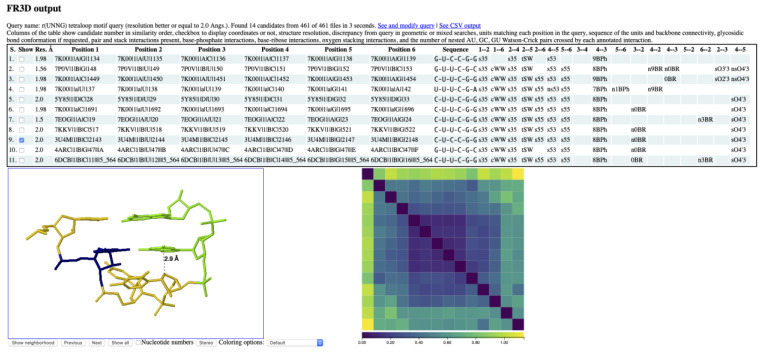
**A WebFR3D symbolic query for r(nU_2_NNGn) Z-turn motifs.** (**Top**) Panel showing the query constraints. (**Bottom**) Partial view of the WebFR3D result panel showing the first 12 instances, the tetraloop of the 3U4M structure, and a heat map showing the 14 by 14 matrix of mutual geometric discrepancies between the instances. The 2.9 Å lp…π contact between the ribose O4′ and the guanine C2 atom is visualized by a dashed line. This search shows that imposing a trans-Sugar…Watson-Crick (tSW) U_2_•G_5_ pair (yellow box) and nucleotides that are consecutive in the (n, n + 1) or (5′ to 3′) direction (“next” in blue boxes) is sufficient to retrieve fourteen r(nUNNGn) folds with the Z-turn characteristics discussed in Section 3.4 and present in the 3.230 representative set of structures [67]. However, a more stringent query is needed to discard problematic structures from this and lower resolution ensembles (Figure S3). Use the following link to retrieve the results of this search: http://rna.bgsu.edu/webfr3d/Results/62c6809102051/62c6809102051.html (accessed on 1 May 2022).

To explore the X-ray and cryo-EM structures of r(U_1_NCG_4_) tetraloops, we used the WebFR3D server to extract a set of instances of r(UNNG) motifs from structures with resolutions ≤ 2.0 Å [5]. The search involves an r(nU_2_NNG_5_n) sequence with a tSW U_2_•G_5_ base pair (Figure 5). The “3.230” representative set of structures (March 2022) with a resolution ≤ 2.0 Å was used [67]. The search led to the 14 hits described below. This structural set comprises eight r(UNCG) X-ray structures, the six other tetraloop structures originating from the 7K00 *Escherichia coli* ribosome cryo-EM structure at 1.98 Å resolution [68]. This ensemble of 14 structures consists of ten r(UUCG), two r(UACG), and two r(UCCG) motifs. With resolutions < 3.0 Å, we found only a single occurrence of an r(UGCG) loop in a *Trypanosoma cruzi* cryo-EM structure (PDBid: 5T5H; res.: 2.54 Å) with a reasonably good electron density map [69] suggesting that this sequence seldom folds as a Z-turn. The third position of r(UNNG) Z-turns can also be a U as discussed in Section 2.1 (Figure 1d) and observed in the 6CK5 riboswitch structure at 2.49 Å resolution [70] or an A as in the 2.7 Å resolution 6YWS ribosomal structure [71].

**Figure 6:**
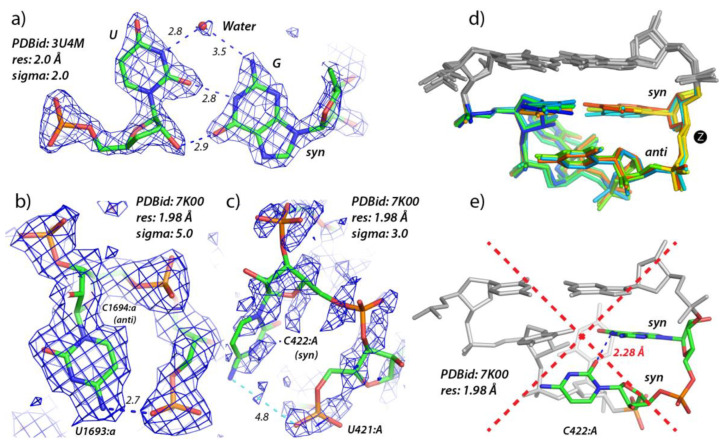
**r(UUCG) structural details derived from X-ray and cryo-EM.** (**a**) A tSW U_2144:B_•G_2147:B_ pair that is part of an r(UUCG) tetraloop showing a water molecule bridging an amino and an imino Watson-Crick site (PDBid: 3U4M). (**b**,**c**) Views of the interaction are shown in Figure 1d for two tetraloops extracted from the 7K00 cryo-EM structure. In (**c**), the density is poor at a 3.0 sigma level and the C422:A residue has been modelled in *syn* leading to a loss of an important interaction shown in (**b**) where the density at a higher 5.0 sigma level is unambiguous. Note that X-ray and cryo-EM density sigma levels are not equivalent. (**d**) Backbone superimposition of the five high-quality 5Y85, 3U4M, 7KKV, 4ARC, and 7K00 r(U_1692:a_UCG) Z-turns mentioned in the text. The superimposition was made by using Chimera [73]. (**e**) View of the 7K00 r(U_420:A_UCG) tetraloop with the cytosine in *syn* and an unrealistic 2.28 Å hydrogen-bond contact [66]. This short contact reveals major modelling issues in regions of a 1.98 Å resolution cryo-EM structure and explains why this tetraloop structure must be discarded.

A closer examination of the eight high-resolution X-ray structures (≤2.0 Å) reveals that the electron densities for the 6DCB and to a lesser extent the 5OB3 tetraloops are of poor quality as also implied by some backbone irregularities, the absence of modeled water molecules, and by the fact that the bottom cytosine residue of the 5OB3 adopts an irregular *syn* conformation. Therefore, these two structures were discarded from the ensemble and the focus was placed on the six X-ray structures 5Y85, 3U4M, 7KKV, 4ARC, 7EOG, and 7P0V with modeled water molecules. The 3U4M structure presents the best defined experimental electron densities, as visualized with Coot [72]. There, as well as in 7KKV and 7P0V, a water molecule in the U•G cleft of the tSW pair could be accurately modeled (Figure 6a). This water molecule is certainly at the origin of the NOESY spectra cross peak signal observed between water and the first uridine of the loop as detailed in Section 2.1. Although the 7EOG model has the best resolution (1.50 Å) of the set, the electron density maps are less well defined, and no water molecule was modeled in the density that is visible in the U•G cleft of the tSW pair. Thus, we discarded 7EOG from our validated structural ensemble.

Among the six r(UNCG) loops found in the 1.98 Å resolution 7K00 cryo-EM structure, the best-defined densities are those of r(U_1692:a_UCG) (Figure 6b) and to a lesser extent of the r(U_343:A_ACG) loop. The former tetraloop is the only match to the 3U4M X-ray (2.0 Å) structure in terms of the quality of both the model and the experimental data. The U•G bridging water molecule is visible in these two tetraloops. On the other hand, the r(U_1135:A_CCG), r(U_1450:A_CCG), and r(U_138:a_UCG) loops feature poor densities as shown by the PDB deposited map visualized with Coot [72]. Finally, the r(U_420:A_UCG) loop has a (C_423:A_)O2…N1(G_424:A_) distance of 2.28 Å because C_423:A_ is unduly modeled in *syn* (Figure 6c).

At this stage, a word of caution is needed. Since these structures were deposited by experienced researchers, it is reasonable to trust the data within the limits of the claimed resolution. However, numerous studies reported that blindly trusting deposited PDB structures can lead to serious data interpretation flaws [66,74,75,76,77,78]. Occasionally, as mentioned above, it is required to inspect the experimental density maps to correct inconsistencies that were not perceived by the authors of the structures. For instance, without careful inspection of electron densities, one could be inclined to consider that the *syn* conformation of the 3rd residue of the 5OB3 tetraloop and other minor structural deviations are the result of natural hairpin dynamics. However, they more likely result from poor local modeling due to an incomplete refinement process combined or not with insufficient experimental data [74,75,78,79,80,81].

Finding structural inconsistencies in a structural ensemble is tedious work. It can be realized, as we detailed above, on small structural ensembles through careful examination of experimental data to validate or invalidate the model. However, for the larger structural ensembles obtained for searches with resolutions ≤ 3.0 Å, this is an impossible task. Thus, more constraints need to be added to filter out inappropriate structures. A search involving resolutions ≤ 2.0 Å with the Figure 5 criteria generates 14 hits, while the same search using the Figure S3 criteria that add an nt4-nt5 sO4′3 constraint and an *anti* constraint on nt3 and nt4 generates 7 hits closely matching our manually curated ensemble. Similarly, a search involving resolutions ≤ 3.0 Å with the Figure 5 criteria generates 82 hits, while the same search using the Figure S3 criteria generates 49 hits.

The water molecule in the U•G cleft might also be considered as part of the r(UNCG) signature (Figure 6a). However, WebFR3D does not currently allow solvent molecule searches. Additionally, including a solvent constraint would result in a low number of hits given that water molecules are only present in a subset of high-resolution structures. Yet, the presence of this water molecule should be considered a hallmark of high-quality experimental data associated with accurate modeling.

### 3.5. Finding Unusual r(GNNA) Z-Turns

**Figure 7:**
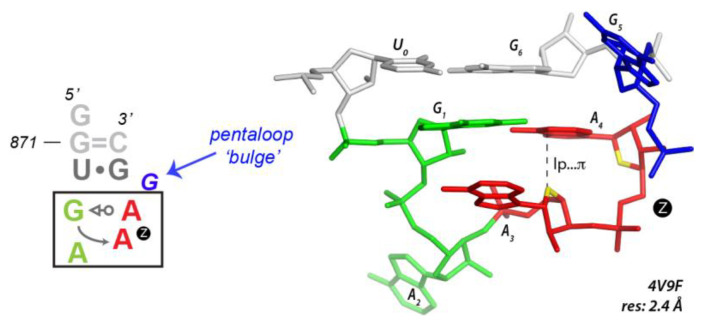
**An r(G_1_AAAg) pentaloop that adopts a Z-turn rather than an expected U-turn.** The Z-step is marked by a circled “Z” in both the secondary and tertiary structure of the ribosomal H35a tetraloop of the *Haloarcula marismortui* large ribosomal subunit (PDBid: 4V9F). This motif is conserved in all known ribosome structures. It can be considered as a “shapeshifting” motif exemplifying the ability of an r(GAAA) loop to switch from U-turns to Z-turns in specific environments [13,31]. Note that a U-turn is characterized by an s3OP2 phosphate…π contact between the first and third residue (not shown), while Z-turns are characterized by sO4′3 between the 3rd and 4th residue and Z_anti_-turns by sO4′5 between the 3rd and 4th residue (Figure S5).

WebFR3D can be used to search regular r(UNCG) Z-turns but can also be used to explore the sequence variability of these structural motifs. In preceding studies [13,31], we identified loops with r(GAAA) sequences that adopt a Z-turn conformation with a tSW G•A pair (Figure 7). This was surprising since most of the r(GAAA) or r(GNRA) tetraloops are known to adopt a U-turn conformation. Such conformations are retrieved by WebFR3D when the r(nGNNAn) sequence, tSW G•A pair, and nt4-nt5 sO4′3 constraints are imposed (Figure S4). WebFR3D also isolated the r(GACA) sequence (a non-GNRA sequence; Figure S4) that has the ability to fold as a Z-turn in the 2.2 Å resolution 7OF0 human mitochondrial ribosome [82] and an r(GUGA) Z-turn in the 1.3 Å resolution 4LGT structure [83]. These r(GAAA), r(GUGA), and r(GACA) Z-turns occur all at the same location in the core of the large ribosomal sub-unit [13,31]. It is important to note that to find these motifs, no tetraloop closing base pair constraint must be imposed given that most if not all of them are pentaloops with a 5th bulging residue. We call this U-to-Z tetraloop motif transition a shapeshifting process that is similar in spirit to the flipons described by A. Herbert. Flipons define a class of sequences capable of forming either left or right-handed helical structures [84,85].

### 3.6. UNNG versus CNNG Z-Turns: Isosteric or Not?

It is appreciated that U•G and C•G tSW pairs that comprise a Y•G pair O2…N1 hydrogen bond (see Figure 1c) are isosteric [86]. As such it seems reasonable to assume that r(UNNG) and r(CNNG) sequences would be equally favored. However, it is observed that r(UNNG) are more represented than r(CNNG) tetraloops [13]. This is reflected by WebFR3D searches that identify 56 instances of r(UNNG) versus 14 instances of r(CNNG) Z-turns at resolutions ≤ 3.0 Å.

Although the observed Z-turn structures are isostructural, we propose a hypothesis that could explain the underrepresentation of r(CNNG) tetraloops. When cytosine and guanines are close in space, they tend to form C=G pairs and this results in the formation of a Z_anti_-turn di-loop [13] with the 4th guanine in *anti* rather than *syn* while the ribose head-to-head conformation is preserved. However, in Z_anti_-turns, the sO4′5 lp…π contact prevails given the *anti* conformation of the 5′-nucleotide. This Z_anti_-turn structure seems more constrained and competes with the formation of an r(CNNG) Z-turn. We found 23 r(CNNG) Z_anti_-turns at resolutions ≤ 3.0 Å. It is interesting that these Z_anti_-turns are still isosteric with Z-turns. They keep the head-to-head ribose orientation in the NpG step, the sO4′3 is replaced by a sO4′5 contact, and the second base is bulged out. It could be argued that Z_anti_-turns with a U_1_•G_4_ cWW pair could also compete with the canonical r(UNNG) Z-turns. Only one such r(UUCG) Z_anti_-turn has been found, in the 6ERI chloroplast ribosome structure at 3.0 Å resolution [87].

The Figure S5 search expands on the number of sequences that can form Z_anti_-turns characterized previously [13]. At resolutions ≤ 3.0 Å, we found 32 examples of Z_anti_-turns that involved the r(CNNG) sequence described above, but also r(UNNA), r(UUCG), and r(GNNC) sequences. Only two of them with an r(CUUG) and an r(UUCG) is closed by a Watson-Crick pair and can be considered as tetraloop hairpins (PDBid: 6AZ3; res. 2.5 Å and PDBid: 7P7Q; res. 2.4 Å). However, the fact that these structures are few, and most of them are located in ribosomes of medium resolution, calls for caution and needs confirmation.

## 4. Discussion

### 4.1. Outliers: Validating, Correcting, or Discarding

An exploratory data analysis of the WebFR3D results is a good way to spot outliers for a given set of structures. A rough identification of outliers may be based on the heatmaps provided by the WebFR3D searches (Figure 5 and Figures S1–S5) that allow rapid identification of structural variations, which can then be individually inspected. These outliers may correspond to rare but real conformations of a given motif or to a locally deficient model based on poorly interpreted experimental data as detailed in Section 3.4. When outliers are identified, it is advised to verify if the model agrees with experimental data and with current knowledge to decide if the explored structural fragment should be categorized as a new conformation, excluded from the dataset, or corrected.

While correcting the structures seems the best option, choosing this process depends on the quality of the available electron density maps. When experimental data are of poor quality, it is not advised to attempt such a correction, and discarding the structure seems a better choice. However, when one is interested in using a structure for initiating MD simulations, it is advised to check the structures and eventually correct any visible flaws [78].

### 4.2. Use of Structural Signatures for MD Simulations

If one is concerned by the integrity of an r(UNCG) structural model derived from MD simulations [51], the WebFR3D constraints defined in Figure S3 should be monitored. Moreover, additional structural features should be examined like the Figure 1d cytosine…ribose interaction that is also part of the structural signature of an r(UNCG) loop. The variations in ribose puckers and dihedral angles also need to be scrutinized. Moreover, the presence of a water molecule in the cleft of the tSW U•G pair (Figure 6a) could be monitored since this water might be considered as part of the r(UNCG) loop signature and should display long residency times as suggested by NMR data. The question remains whether MD simulations should reproduce this solvation feature. The answer is probably yes. However, these solvation features may be less stable than the tSW base pairing.

Recently, several of these contacts were monitored in MD simulations including a labile 0BPh contact involving the G of the first stem base pair [54,59]. Such C-H…O contact [88] are very subtle and difficult to model although recent parameterization efforts involving a modification of vdW radii improved the sampling of the loops. Unfortunately, the conclusions of these studies reveal that we are currently far from mastering all interactions that are essential for modeling the apparently “simple” tetraloop systems [54,59].

### 4.3. WebFR3D Limitations and Strategy

Additional features are regularly being added to the WebFR3D search interface but, understandably, not all conceivable search constraints are available. For example, one cannot use the presence or absence of a solvent molecule as a search constraint, nor can one constrain a search by sugar pucker or backbone conformation. Instead, these features must be evaluated on a case-by-case basis in the relatively small set of instances returned by searches targeting high-resolution structures.

A recurrent issue in searches of biological 3D structures is over-constraining the search and missing instances of interest because they happen to lack one or more of the specified criteria. Sometimes, one should use as few symbolic constraints as possible to get the instances of interest; a search that insists on all idealized features of a motif may return no or very few results! For avoiding such issues, one can widen the search criteria by allowing “near” annotations. For example, when searching for an r(UNNG) turn and using a U•G tSW base pair constraint, one may wish to allow both “near” and “true” tSW base pairs by typing “tSW ntSW UG” or “n+tSW UG” in the appropriate WebFR3D yellow search box. This will return a wider range of instances, which can be evaluated manually. Finally, if one already has an instance of a desired motif, one can search for geometrically similar instances up to a user specified geometric discrepancy value, while still imposing a minimal set of symbolic constraints, and thus avoid over-constraining a search based on incorrect guesses about how instances of interest will vary.

## 5. Conclusions

Our WebFR3D lp…π constraint implementation was demonstrated to be effective in retrieving instances of all categories of lp…π contacts between consecutive and non-consecutive nucleotides in RNA. As such, we confirmed preceding findings that assessed that basically all 16 NpN combinations can form Z-steps with the current exception of CpC steps and showed without surprise that the most represented ones are CpG Z-steps. The current implementation of WebFR3D is also able to retrieve all kinds of motifs including a phosphate…π contact.

We were also able to successfully retrieve all types of Z-turns described elsewhere [13]. In addition, through the fine tuning of structural constraints, we could eliminate from an ensemble of r(UNCG) structures with resolutions < 2.0 Å, locally poorly resolved structures and refine the r(UNCG) signature to provide better comparison points for structural and molecular dynamics (MD) simulation studies.

WebFR3D proved also effective in being able to search for r(NNNN) sequences that fold as Z-turns, that is to search for all the sequences able to adopt a given 3D shape. For instance, we retrieved r(GAAA) motifs that are isostructural to r(UNCG) Z-turns. Another highlighted example is related to r(CNNG) sequences that can fold as Z-turns and are therefore almost completely isostructural to r(UNCG) loops. Alternatively, they can fold as Z_anti_-turns and adopt a structure with a C_1_=G_4_ Watson-Crick base pair and a G_4_ nucleotide in *anti* rather than in *syn*. These loops are also isostructural with r(UNCG) loops.

Such examples demonstrate that searching by shape is complementary to searching by sequence and that when studying an RNA for which 3D structures are not available, one should consider the multiple shapes a given sequence can adopt by exploring their diversity with tools such as WebFR3D.

## 6. Methods

**WebFR3D search tool:** WebFR3D is the online implementation of FR3D, a general-purpose RNA motif search tool [4,5]. WebFR3D makes it possible to search for RNA motifs of one to over a dozen nucleotides in different search modes. In a geometric search, the user specifies a list of RNA nucleotides from a 3D structure from the Protein Data Bank and a set of structures to search, and WebFR3D is guaranteed to find all matches up to a user-specified tolerance called the discrepancy cutoff. Geometric searches can be augmented with symbolic constraints to require certain base identities, pairwise interactions, or chain continuity constraints. Some motifs can be described entirely by symbolic constraints, and WebFR3D makes it possible to search for those based on the constraints alone. WebFR3D has pre-computed annotations of RNA base pairs, base stacking, base-backbone, and other interactions for all RNA-containing 3D structures in PDB. For statistical surveys, one can avoid the redundancy inherent in the 3D structure database by searching a representative set of 3D structures, at a chosen resolution threshold [67].

**Attribution of a nucleobase 3′ and 5′-face:** For each standard RNA base, the BGSU RNA pipeline [4,5] calculates the geometric center of the heavy (non-hydrogen) atoms of the base, weighting each heavy atom equally. From this point, the normal vector to the base is oriented to point toward the 3′-direction in a regular RNA helix. We refer to that nucleobase face as the 3′-face; the opposite face becomes the 5′-face. This convention was introduced earlier and is also used to annotate base-base stacking by FR3D [4].

**lp…π search:** For each nucleotide pair, the relative locations of the base of the first nucleotide and the backbone oxygen atoms of the second are computed in the following way. For each of the oxygen atoms in the phosphate backbone of the second nucleotide, the vertical coordinate along the normal vector of the first base, called z, is determined. For |z| ≤ 3.5 Å, the oxygen atom is projected onto the plane of the first base; for an lp…π contact, it must lie inside a nucleobase ring. If more than one oxygen atom projects inside a ring, we keep the oxygen atom with the smallest |z| value. We annotate an oxygen stacking interaction according to the oxygen atom and the face of the first base, producing a string such as sO4′3 when the O4′ atom stacks with the 3′-face. See Figure S6 for plots of the projected oxygen atoms having minimal |z| values.

When the criteria for an lp…π contact are not met, we check for “near” stacking interactions to soften the boundaries between contacts that have all the desired properties and contacts that are nowhere close to sO stacking.

Case 1: An oxygen has 3.5 Å < |z| ≤ 3.6 Å and projects inside a ring; we keep the oxygen atom with the smallest |z| value.

Case 2: An oxygen has |z| ≤ 3.5 Å and projects outside the ring(s) but within an ellipse around a ring which we describe now.

For each standard RNA base ring, the ellipse that comes closest to the five or six atoms on a ring is drawn (note that the eccentricities of the ellipses are small but not zero). Then we expand the ellipse to 0.3 Å beyond the corners of the ring, as shown in green in the bottom panel of Figure S6. Among such oxygen atoms, we annotate the one that is closest to the geometric center of the base, to produce an annotation such as nsO4′3 where “n” stands for “near”. For searching both regular and near instances, one may use a text string such as n+sO4′3. Figure S6 shows the projected oxygen atoms in sO and near sO (nsO) interactions. This methodology resembles that described in [12].

## Data Availability

Not applicable.

## Supplementary Materials

The following supporting information can be downloaded at: https://www.mdpi.com/article/10.3390/molecules27144365/s1, Figure S1: A WebFR3D symbolic query for finding all lp…π contacts; Figure S2: A WebFR3D symbolic query for finding NpN Z-steps; Figure S3: A strict symbolic query for refining Z-turn searches with a 2.0 Å resolution limit; Figure S4: WebFR3D finds GNNA Z-turns; Figure S5: WebFR3D finds Z_anti_-turns that fold similarly to Z-turns; Figure S6: WebFR3D hits as visualized over the four nucleobases with a sO search at 3.0 Å resolution.
