# Supplementary material for: Lone Pair…π Contacts and Structure Signatures of r(UNCG) Tetraloops, Z-Turns, and Z-Steps: A WebFR3D Survey
Source: Molecules. 2022 Jul 7;27(14):4365. doi: 10.3390/molecules27144365 (PMC9323530; doi:10.3390/molecules27144365)
Supplement: Supplementary file 1 [file molecules-27-04365-s001.zip › molecules-1758873-supplementary.pdf]

Supplementary Material

Lone pair…π contacts and structure signatures of r(UNCG) tetraloops, Z-turns and Z-steps: A WebFR3D survey

Craig L. Zirbel[1] and Pascal Auffinger[2*]

[1.] Department of Mathematics and Statistics, Bowling Green State University, Bowling Green, OH 43403, USA;
[2.] Architecture et Réactivité de l'ARN, UPR 9002, Université de Strasbourg, Institut de Biologie Moléculaire et Cellulaire du CNRS, 67084 Strasbourg, France;

Tel: +33 388417049; Fax: +33 388602218;
Email: p.auffinger@ibmc-cnrs.unistra.fr;

Contents:

Figure S1: A WebFR3D symbolic query for finding all lp…π contacts.

Figure S2: A WebFR3D symbolic query for finding NpN Z-steps.

Figure S3: A strict symbolic query for refining Z-turn searches with a 2.0 Å resolution limit.

Figure S4: WebFR3D finds GNNA Z-turns.

Figure S5: WebFR3D finds Z_{anti}-turns that fold similarly to Z-turns.

Figure S6: WebFR3D hits as visualized over the four nucleobases with a sO search at 3.0 Å resolution.

References

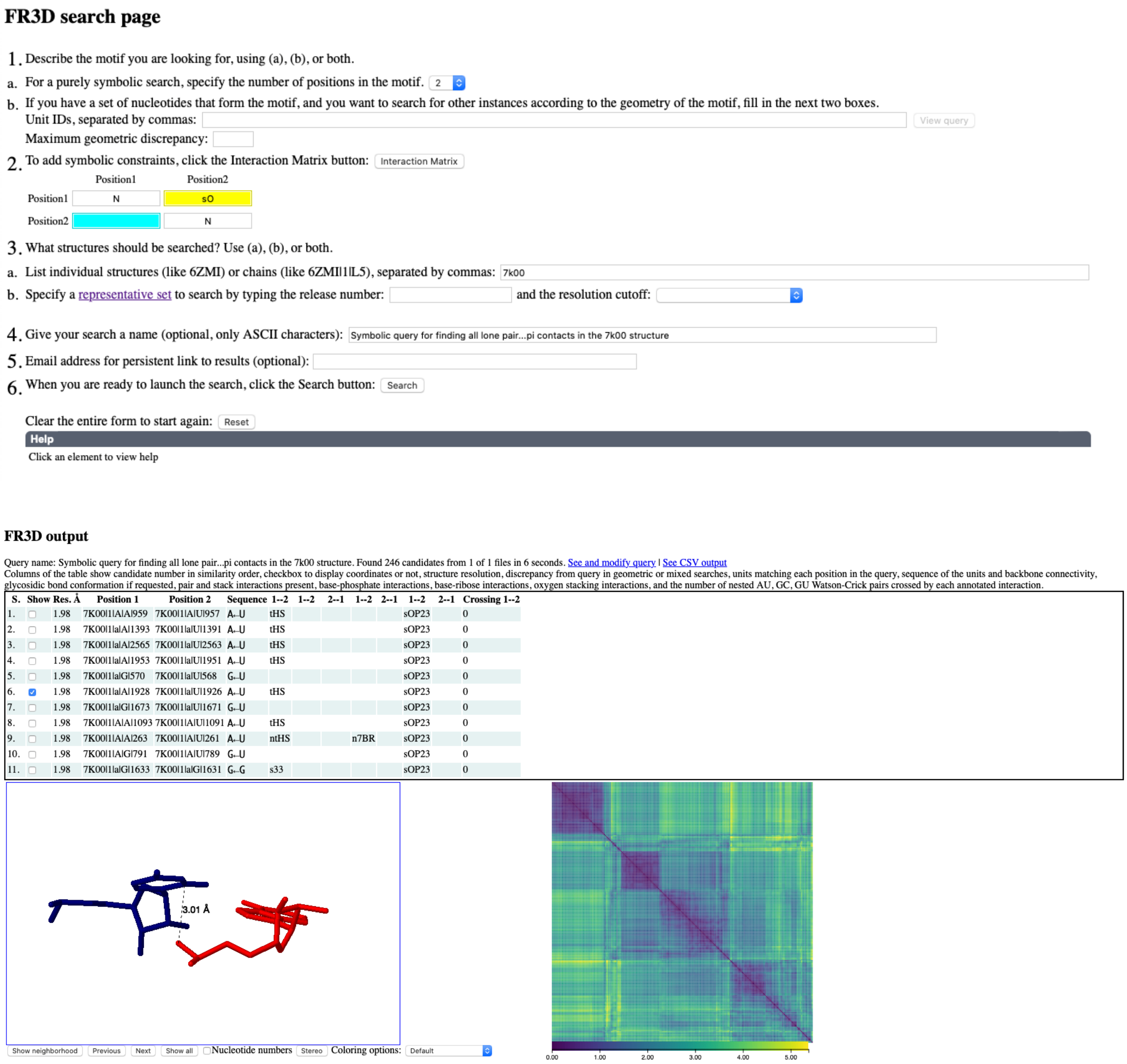

Figure S1: A WebFR3D symbolic query for finding all lp…π contacts. (Top) The search panel using the text string "sO" retrieves all instances of a stacking of a backbone oxygen atom (OP1, OP2, O2', O3', O4', and O5') from nt1 with a nucleobase of nt2. The string "sO" expands to "sO3 sO5" which expands to include all backbone oxygens. (Bottom) Partial view of the WebFR3D result panel showing a sOP23 contact (OP2 atom stacking with the 3'-face of a uridine) present in the 7K00 *Escherichia coli* 70S cryo-EM structure [1]. The shortest lp…π contact visualized by a dashed line involves the (U)C2 atom. Note that 246 potential lp…π contacts are identified in 7K00. Use the following link to retrieve the results of this search:
http://rna.bgsu.edu/webfr3d/Results/6287e7c307b40/6287e7c307b40.html

(last accessed the 6th of July 2022)

FR3D search page

1. Describe the motif you are looking for, using (a), (b), or both.

a. For a purely symbolic search, specify the number of positions in the motif. 2

b. If you have a set of nucleotides that form the motif, and you want to search for other instances according to the geometry of the motif, fill in the next two boxes.
Unit IDs, separated by commas: View query
Maximum geometric discrepancy:

2. To add symbolic constraints, click the Interaction Matrix button: Interaction Matrix

	Position1	Position2
Position1	N anti	sO4'3
Position2	next	N syn

3. What structures should be searched? Use (a), (b), or both.

a. List individual structures (like 6ZMI) or chains (like 6ZMI|1|L5), separated by commas: 7k00

b. Specify a representative set to search by typing the release number: and the resolution cutoff:

4. Give your search a name (optional, only ASCII characters): Symbolic query for finding all Z-steps in the 7k00 structure

5. Email address for persistent link to results (optional):

6. When you are ready to launch the search, click the Search button: Search

Clear the entire form to start again: Reset

Help

Click an element to view help

FR3D output

Query name: Symbolic query for finding all Z-steps in the 7k00 structure. Found 11 candidates from 1 of 1 files in 2 seconds. See and modify query | See CSV output
Columns of the table show candidate number, radio button to display coordinates or not, discrepancy from query in geometric or mixed searches, units in each position of the motif, pair and stack interactions, base-phosphate interactions, base-ribose interactions, and crossing number for each interaction.

S	Show	Res Å	Position 1	Position 2	Sequence	Orient 1	Orient 2	Chi 1	Chi 2	1--2	1--2
1.	☐	1.98	7K00|1|a|A|2266	7K00|1|a|A|2267	A-A	anti	syn	-136	70	ns53	sO4'3
2.	☑	1.98	7K00|1|A|G|721	7K00|1|A|G|722	G-G	anti	syn	-146	63	s53	sO4'3
3.	☐	1.98	7K00|1|a|A|1020	7K00|1|a|A|1021	A-A	anti	syn	-133	79	s53	sO4'3
4.	☐	1.98	7K00|1|a|C|527	7K00|1|a|A|528	C-A	anti	syn	-145	71	s53	sO4'3
5.	☐	1.98	7K00|1|A|U|982	7K00|1|A|A|983	U-A	anti	syn	-136	72	s53	sO4'3
6.	☐	1.98	7K00|1|a|U|932	7K00|1|a|A|933	U-A	anti	syn	-140	83	s53	sO4'3
7.	☐	1.98	7K00|1|a|G|2307	7K00|1|a|G|2308	G-G	anti	syn	-126	75	s53	sO4'3
8.	☐	1.98	7K00|1|A|C|345	7K00|1|A|G|346	C-G	anti	syn	-119	69	s53	sO4'3
9.	☐	1.98	7K00|1|a|C|1694	7K00|1|a|G|1695	C-G	anti	syn	-132	57	s53	sO4'3
10.	☐	1.98	7K00|1|a|C|1451	7K00|1|a|G|1452	C-G	anti	syn	-108	64		sO4'3
11.	☐	1.98	7K00|1|a|U|1141	7K00|1|a|A|1142	U-A	anti	syn	-104	74		sO4'3

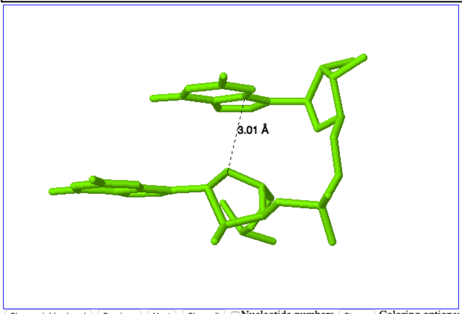

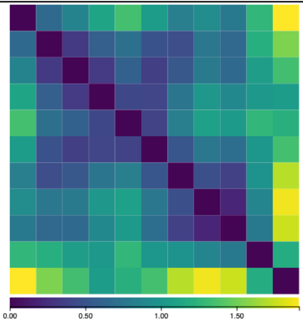

Show neighborhood | Previous | Next | Show all | Nucleotide numbers | Stereo | Coloring options: Default

Figure S2: A WebFR3D symbolic query for finding NpN Z-steps. (Top) The search panel using the text string (sO4'3) indicates that all instances of a stacking of a O4' atom with the 3'-face of a nucleobase will be retrieved. To gather Z-steps, it is also necessary to specify that the nucleotides are consecutive ("next" condition), that the O4' ribose of the 5'-nt stacks with the 3'-face of the 3'-nt that is in *syn*. In a few instances, the 3'-nt can also be *anti*. When this constraint is given the search retrieves Z_{anti}-steps [2]. (Bottom) Partial view of the WebFR3D result panel showing a GpG Z-step in the 7K00 structure (note the head-to-head orientation of the riboses; see also **Figure 3b**). The lp…π shortest contact distance that is visualized by a dashed line involves the (G)C4 atom. Note that eleven potential NpN Z-steps are identified in the 7K00 *Escherichia coli* 70S structure. Use the following link to retrieve the results of this search:
http://rna.bgsu.edu/webfr3d/Results/627e8a7b5856f/627e8a7b5856f.html

(last accessed the 6th of July 2022)

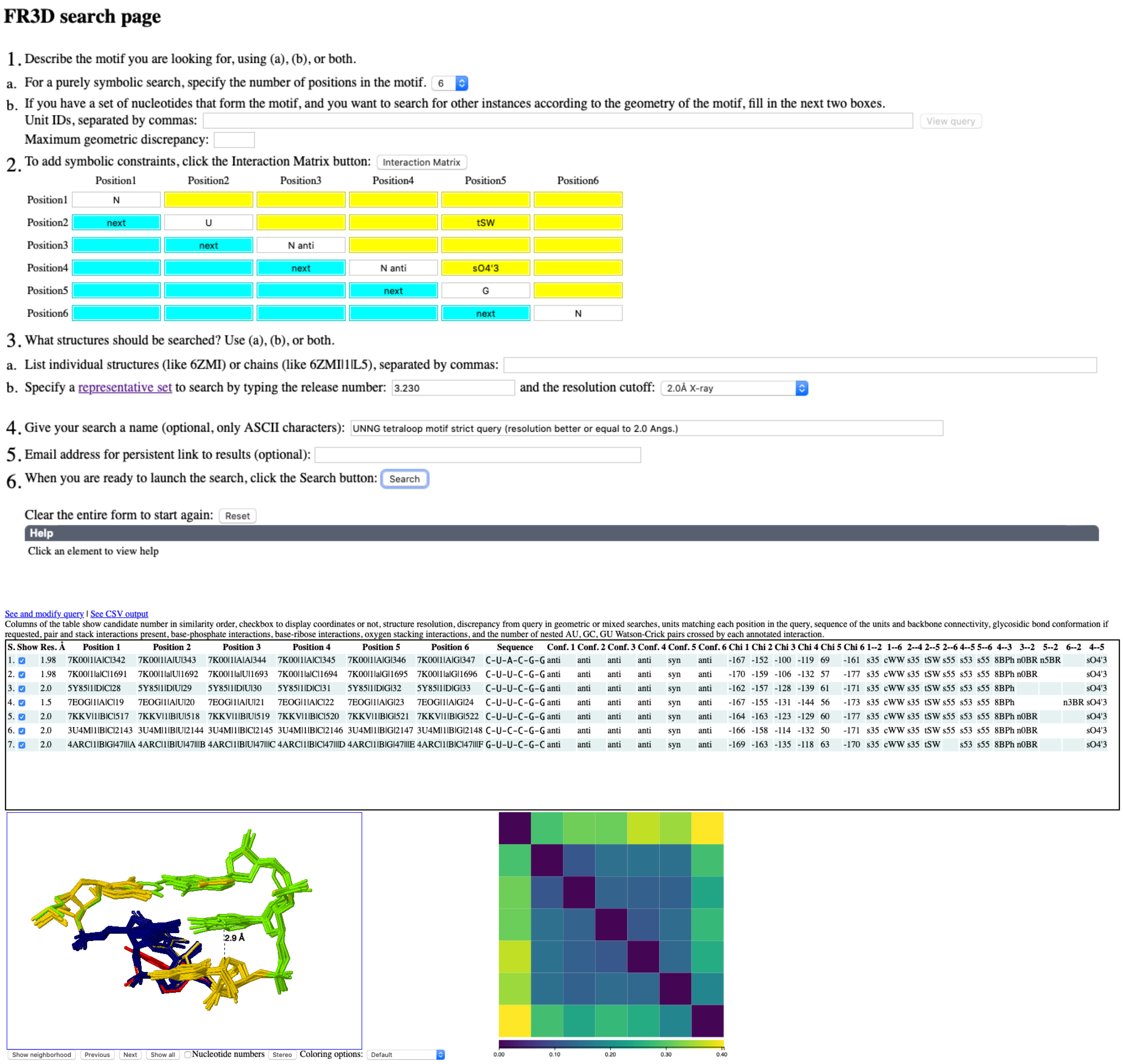

Figure S3 A strict symbolic query for refining Z-turn searches with a 2.0 Å resolution limit. (Top) WebFR3D search page with more stringent search criteria than those used in **Figure 5.** (Bottom) Partial view of the WebFR3D result panel showing an overlay of seven structures. The 2.9 Å lp…π contact shown in **Figure 5** for 3U4M is visualized by a dashed line. The rationales for applying more stringent search criteria are described next. By examining the result panel of **Figure 5**, we noted that four of the 14 turns do not display strict Z-step lp…π contacts. Therefore, we added a nt4…nt5 sO4'3 constraint. This narrows the search down to ten hits. Two additional Z-turns display a s33 stacking between nt4 and nt5 instead of the more common r(UNNG) s53 stacking pattern. This reveals that the bottom nucleotide is in *syn* and not *anti*. By discarding these two structures through imposing an *anti*-conformation to the 3rd tetraloop residue, the structural ensemble comprises now eight elements. A further examination of the **Figure 5** data reveals that the 2nd residue of 6DCB is in "Intermediate *syn*" and as noted in **Section 3.4** of the main text, the local electron density associated with this tetraloop is of poor quality motivating the 6DCB exclusion from the set of structures. Use the following link to retrieve the results of this search:

http://rna.bgsu.edu/webfr3d/Results/6287e67695a70/6287e67695a70.html

(last accessed the 6th of July 2022)

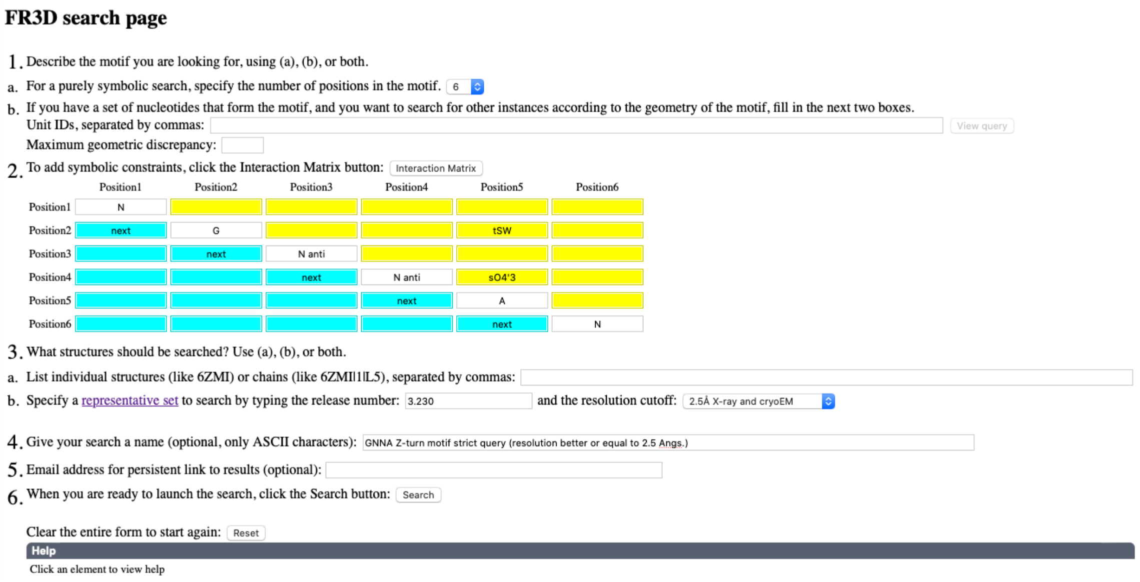

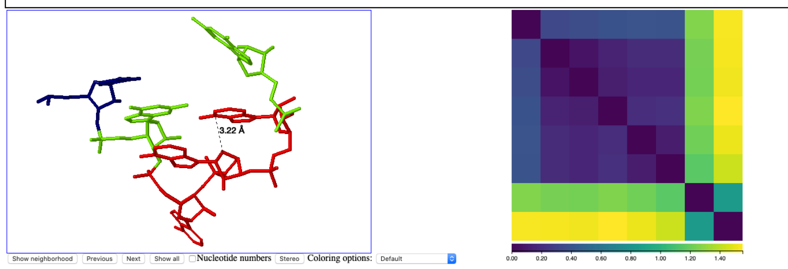

FR3D output

Query name: GNNA Z-turn motif strict query (resolution better or equal to 2.5 Angs.). Found 8 candidates from 933 of 933 files in 14 seconds. See and modify query | See CSV output
Columns of the table show candidate number, radio button to display coordinates or not, discrepancy from query in geometric or mixed searches, units in each position of the motif, pair and stack interactions, base-phosphate interactions, base-ribose interactions, and crossing number for each interaction.

S	Show	Res. Å	Position 1	Position 2	Position 3	Position 4	Position 5	Position 6	Sequence	Orient 1	Orient 2	Orient 3	Orient 4	Orient 5	Orient 6	Chi 1	Chi 2	Chi 3	Chi 4	Chi 5	Chi 6	1--2	1--6	2--4	2--5	2--6	4--5	5--6	4--3	5--4	4--3	5--2	5--6	4--5
1.	☐	2.2	7OF0|1|A|C|1989	7OF0|1|A|G|1990	7OF0|1|A|A|1991	7OF0|1|A|C|1992	7OF0|1|A|A|1993	7OF0|1|A|A|1994	C-G-A-C-A-A	anti	anti	anti	anti	anti	anti	-163	-160	-142	-128	102	-172	s35		s35	tSW		ns53			n7BPh		n7BR	n2BR	sO4'3
2.	☑	2.4	4V9F|1|0|U|872	4V9F|1|0|G|873	4V9F|1|0|A|874	4V9F|1|0|A|875	4V9F|1|0|A|876	4V9F|1|0|G|877	U-G-A-A-A-G	anti	anti	anti	anti	anti	anti	-161	-158	-144	-117	98	-155	s35		s35	tSW		ns53					n7BR	n2BR	sO4'3
3.	☐	2.1	4YBB|1|DA|U|779	4YBB|1|DA|G|780	4YBB|1|DA|A|781	4YBB|1|DA|A|782	4YBB|1|DA|A|783	4YBB|1|DA|G|784	U-G-A-A-A-G	anti	anti	anti	anti	anti	anti	-166	-158	-143	-108	98	-156	s35		s35	tSW		ns53					n7BR	n2BR	sO4'3
4.	☐	2.5	6AZ3|1|1|C|962	6AZ3|1|1|G|963	6AZ3|1|1|A|964	6AZ3|1|1|A|965	6AZ3|1|1|A|966	6AZ3|1|1|G|967	C-G-A-A-A-G	anti	anti	anti	anti	anti	anti	-166	-158	-149	-122	102	-161	s35		s35	tSW		ns53			n7BPh			n2BR	sO4'3
5.	☐	2.3	6S0Z|1|A|A|824	6S0Z|1|A|G|825	6S0Z|1|A|A|826	6S0Z|1|A|A|827	6S0Z|1|A|A|828	6S0Z|1|A|U|829	A-G-A-A-A-U	anti	anti	anti	anti	anti	anti	-163	-160	-150	-116	97	-152	s35		s35	tSW		ns53					n7BR	n2BR	sO4'3
6.	☐	2.4	7P7Q|1|A|A|818	7P7Q|1|A|G|819	7P7Q|1|A|A|820	7P7Q|1|A|A|821	7P7Q|1|A|A|822	7P7Q|1|A|U|823	A-G-A-A-A-U	anti	anti	anti	anti	anti	anti	-168	-158	-144	-109	102	-133	s35		s35	tSW		s53			n7BPh		n7BR	n2BR	sO4'3
7.	☐	1.3	4LGT|1|E|C|2594	4LGT|1|E|G|2595	4LGT|1|E|U|2596	4LGT|1|E|G|2597	4LGT|1|E|A|2598	4LGT|1|E|G|2599	C-G-U-G-A-G	anti	anti	anti	anti	syn	anti	-167	-155	-126	-136	58	-169	s35	cWW	s35	tSW	s55	s53	s55			n0BR	7BR		sO4'3
8.	☐	2.4	7P7Q|1|A|C|2156	7P7Q|1|A|G|2157	7P7Q|1|A|C|2158	7P7Q|1|A|U|2159	7P7Q|1|A|A|2160	7P7Q|1|A|G|2161	C-G-C-U-A-G	anti	anti	anti	anti	syn	anti	-169	-154	-138	-129	45	-175	s35	cWW	s35	tSW	s55	s53	s55	n9BPh					sO4'3

Figure S4: WebFR3D finds GNNA Z-turns. (Top) To search r(GNNA) Z-turns, we change the base identity in positions 2 and 5 but otherwise impose the same constraints as in **Figure S3**. (Bottom) The search retrieves several instances of r(GNNA) sequences that adopt a Z-turn conformation with an ApA Z-step. Besides, it finds a r(GACA) and a r(GUGA) Z-turn. A sO4’3 contact to the (A)C2 atom is visualized by a dashed line. Use the following link to retrieve the results of this search:
http://rna.bgsu.edu/webfr3d/Results/6280cd2b79770/6280cd2b79770.html

(last accessed the 6th of July 2022)

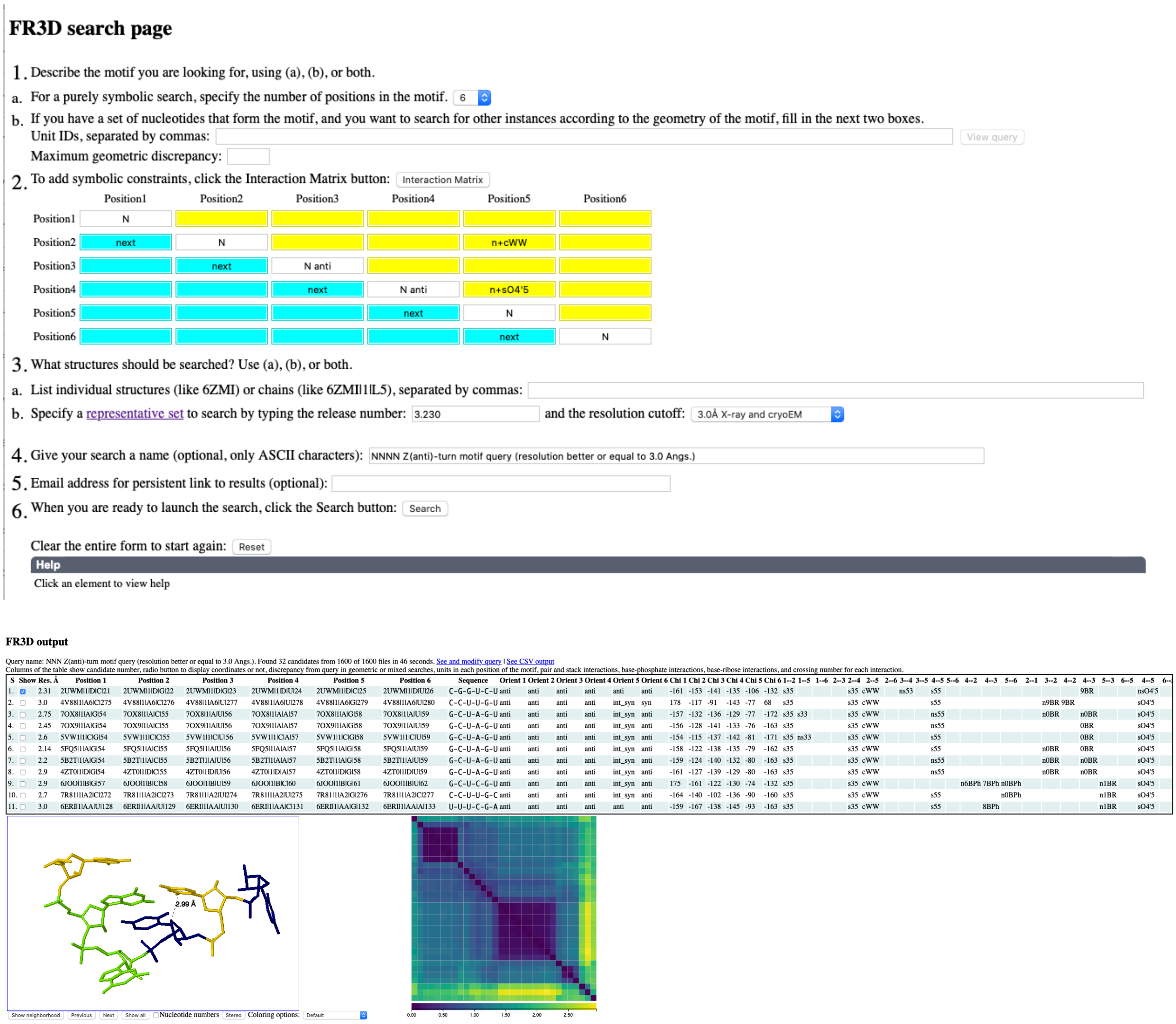

Figure S5: WebFR3D finds Z_{anti}-turns that fold similarly to Z-turns. (Top) To search for r(NNNN) Z_{anti}-turns, we follow the general model of **Figure S3**. However, we need to impose a nt2…nt5 cWW constraint and we need to change the nt4…nt5 sO4'3 to sO4'5 given the *anti* versus *syn* orientation of the 5'-nt. We include near versions of these interactions to retrieve a wider range of instances. (Bottom) A search on structures with resolution ≤ 3.0 Å retrieves 32 instances of Z_{anti}-turns. The structure shown is a surprising r(GGUC) Z_{anti}-turn; the shortest contact distance of the O4' atom with the cytosine ring involves the C2 atom and is shown by a dashed line. To retrieve this contact, we used the "near" syntax, i.e., n+sO4'5 (see **Method section**). Use the following link to retrieve the results of this search:
http://rna.bgsu.edu/webfr3d/Results/628109954b02d/628109954b02d.html

(last accessed the 6th of July 2022)

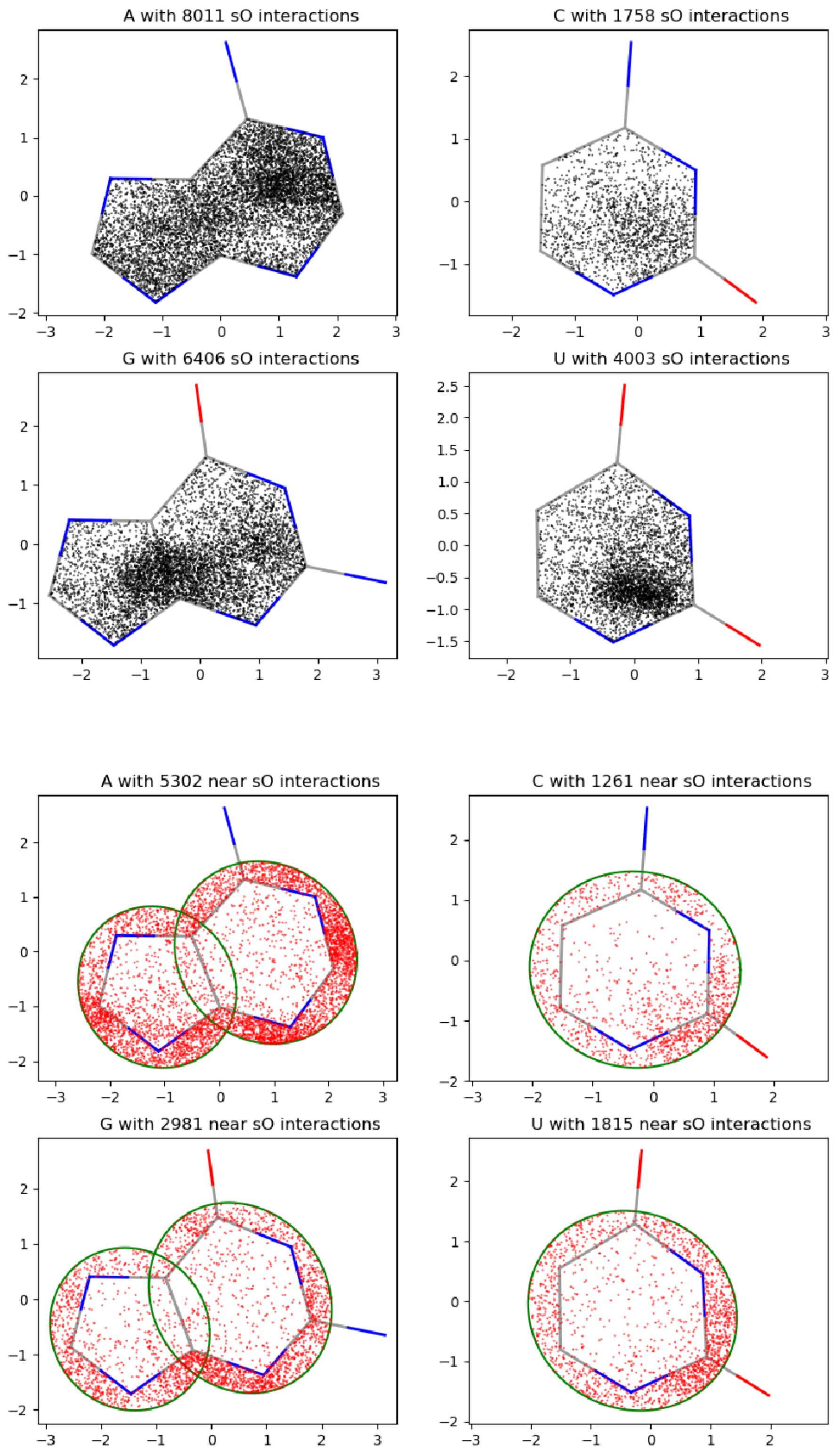

Figure S6: WebFR3D hits as visualized over the four nucleobases with a sO search at 3.0 Å resolution. (Top panels) Oxygen locations projected onto the plane of the base, obtained with a sO search as shown in **Figure S1** for all four nucleobases from an ensemble of structures with resolution ≤ 3.0 Å. (Bottom panels) These panels show the projected oxygen locations in “near” hits obtained with a nsO constraint and explain the working of the “near” keyword.

References

1. Watson, Z. L.; Ward, F. R.; Meheust, R.; Ad, O.; Schepartz, A.; Banfield, J. F.; Cate, J. H., Structure of the bacterial ribosome at 2 Å resolution. *Elife* **2020,** 9.
2. D'Ascenzo, L.; Leonarski, F.; Vicens, Q.; Auffinger, P., Revisiting GNRA and UNCG folds: U-turns versus Z-turns in RNA hairpin loops. *RNA* **2017,** 23, 259-269.
